# In Situ Gelling Systems Using Pluronic F127 Enhance Corneal Permeability of Indomethacin Nanocrystals

**DOI:** 10.3390/ijms21197083

**Published:** 2020-09-25

**Authors:** Noriaki Nagai, Takumi Isaka, Saori Deguchi, Misa Minami, Mizuki Yamaguchi, Hiroko Otake, Norio Okamoto, Yosuke Nakazawa

**Affiliations:** 1 Faculty of Pharmacy, Kindai University, 3-4-1 Kowakae, Higashi-Osaka, Osaka 577-8502, Japan; 1611610016p@kindai.ac.jp (T.I.); 2045110002h@kindai.ac.jp (S.D.); 2033420004w@kindai.ac.jp (M.M.); 2033420005s@kindai.ac.jp (M.Y.); hotake@phar.kindai.ac.jp (H.O.); 2 Okamoto Eye Clinic, 5-11-12-312 Izumicho, Suita, Osaka 564-0041, Japan; eyedoctor9@msn.com; 3 Faculty of Pharmacy, Keio University, 1-5-30 Shibakoen, Minato-ku, Tokyo 105-8512, Japan; nakazawa-ys@pha.keio.ac.jp

**Keywords:** nanoparticles, indomethacin, Pluronic F-127, in situ gelling system, ophthalmic delivery system

## Abstract

We previously designed an ophthalmic dispersion containing indomethacin nanocrystals (IMC-NCs), showing that multiple energy-dependent endocytoses led to the enhanced absorption of drugs from ocular dosage forms. In this study, we attempted to prepare Pluronic F-127 (PLF-127)-based in situ gel (ISG) incorporating IMC-NCs, and we investigated whether the instillation of the newly developed ISG incorporating IMC-NCs prolonged the precorneal resident time of the drug and improved ocular bioavailability. The IMC-NC-incorporating ISG was prepared using the bead-mill method and PLF-127, which yielded a mean particle size of 50–150 nm. The viscosity of the IMC-NC-incorporating ISG was higher at 37 °C than at 10 °C, and the diffusion and release of IMC-NCs in the IMC-NC-incorporating ISG were decreased by PLF-127 at 37 °C. In experiments using rabbits, the retention time of IMC levels in the lacrimal fluid was enhanced with PLF-127 in the IMC-NC-incorporating ISG, whereby the IMC-NC-incorporating ISG with 5% and 10% PLF-127 increased the transcorneal penetration of the IMCs. In contrast to the results with optimal PLF-127 (5% and 10%), excessive PLF-127 (15%) decreased the uptake of IMC-NCs after instillation. In conclusion, we found that IMC-NC-incorporating ISG with an optimal amount of PLF-127 (5–10%) resulted in higher IMC corneal permeation after instillation than that with excessive PLF-127, probably because of the balance between higher residence time and faster diffusion of IMC-NCs on the ocular surface. These findings provide significant information for developing ophthalmic nanomedicines.

## 1. Introduction

Drugs are commonly administered topically to the eye for therapy in ophthalmic diseases, and various approaches for delivering drugs to the eye have been investigated. However, traditional ophthalmic formulations are eliminated from the ocular surface immediately upon instillation because of blinking, the tear reflex, nasolacrimal drainage, poor corneal permeability, lacrimal secretion, and nasolacrimal drainage [1], and the dose reaching the aqueous humor is less than 1% of the instilled dose [2,3,4,5,6]. In previous studies, it was considered that the extension of the precorneal resident time of the drug, and the enhancement of transcorneal penetration can overcome low drug absorption by the eye [7]. Therefore, increasing precorneal drug retention and enhancing the permeability of drugs across the cornea are key to improving low ocular drug bioavailability (BA). Several approaches to extending the precorneal resident time of drugs consist of increasing the viscosity of the dosage form using a viscosity-imparting agent [8]. In addition, the development of ophthalmic drug-delivery systems (DDSs) and novel administration formulations, such as ointments, liposomes, nanoparticles (NPs), microparticles, preformed gels [9], nanostructured lipid carriers, ion-triggered release [10], nanosuspensions, dendrimers, nanocrystals, and pH-sensitive formulations [11,12], were recently reported [12,13,14,15,16,17]. We also designed ophthalmic dispersions containing indomethacin (IMC) nanocrystals (NCs) using bead mill method, and the particle-size frequencies was approximately 100 nm [18,19,20]. Furthermore, we showed that the nanoparticles of IMC induced the multiple energy-dependent endocytosis in the cornea, and caveolae-dependent endocytosis (CME), clathrin-dependent endocytosis (CME), and micropinocytosis (MP) enhanced ocular absorption of IMC from ocular dosage forms [21]. Moreover, we reported that the 1.5% methylcellulose (MC) in situ gelling system was gelled after the instillation, and enhanced contact time with the ocular surface of NCs, resulting in improving the ocular BA [22,23]. Thus, in situ gel (ISG)-incorporating NCs may achieve high ocular drug BA by avoiding rapid precorneal clearance.

Gel systems that are instilled as a solution and transform into a gel at the ocular surface are called in situ gelling systems, and ISGs used as in situ gelling systems usually undergo reversible sol–gel phase transitions [24]. ISGs are polymer solutions that can be administered as a solution before undergoing a phase transition to a semisolid gel when exposed to physiological environments, such as the ocular surface. These gel systems can be triggered by ultraviolet induction, temperature change, solvent exchange, ion interactions, and pH change.

Pluronic F-127 (PLF-127) is a polymer possessing thermoresponsive behavior, and PLF-127 belongs to a family of more than 30 ABA block copolymers. PLF-127 behaves like a nonionic surfactant, due to the amphiphilic nature of its block units. Additionally, the polymer forms a thermoreversible gel in concentrated aqueous solutions, and higher concentration leads to better gel strength [25]. Due to its unique thermoreversible gelation properties, PLF-127 is reported to be well-tolerated, with little irritation and sensitization to the skin and mucous membranes; it was previously used in ocular, rectal, and intranasal formulations [26,27,28]. Moreover, it was known that the PLF-127 exists as liquid before instillation, but changes to a gel on the ocular surface (after instillation), since the gelling of PLF-127 was related to temperature. From the character, the addition of PLF-127 extends the corneal residence time and improves ocular bioavailability [29]. In this study, we attempted to prepare a PLF-127-based IMC-NC-incorporating ISG (IMC-NC/PLF), and we investigated whether the instillation of the newly developed IMC-NC-incorporating ISG using PLF-127 prolonged the precorneal resident time of the drug and improved ocular BA.

## 2. Results

### 2.1. Design of IMC-NC-Incorporating ISG

**Figure 1:**
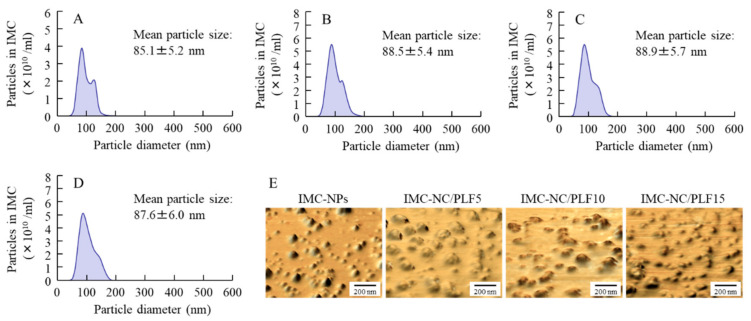
Particle-size frequencies and atomic force microscopy (AFM) images of indomethacin (IMC) in each ophthalmic indomethacin nanocrystal (IMC-NC) formulation. (**A**–**D)** Particle-size frequencies of IMC in (**A**) ophthalmic dispersions containing IMC-NCs (IMC-NPs), (**B**) IMC-NCs with 5 g of Pluronic F-127 (IMC-NC/PLF5), (**C**) IMC-NC/PLF10, and (**D**) IMC-NC/PLF15. (**E**) AFM images of IMC in IMC-NPs, IMC-NC/PLF5, IMC-NC/PLF10, and IMC-NC/PLF15. Compositions of each ophthalmic IMC-NC formulation shown in Table 1. IMC particles in each ophthalmic IMC-NC formulation were 50–170 nm.

**Figure 2:**
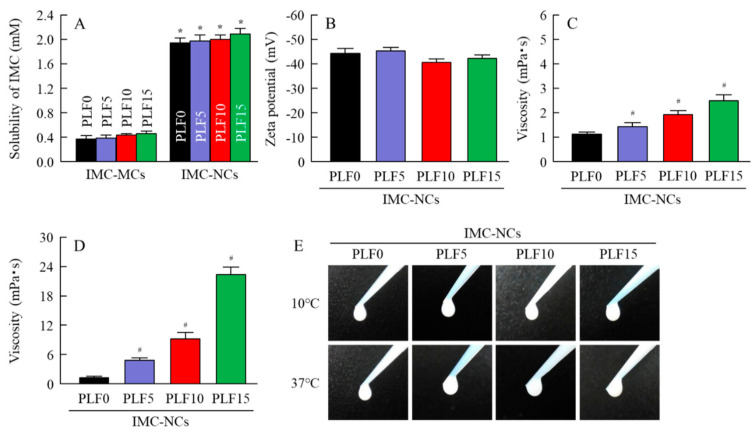
Changes in solubility, zeta potential, and viscosity of each ophthalmic IMC-NC formulation. (**A**) Solubility of IMC in IMC microcrystals (IMC-MCs) and IMC-NCs with Pluronic F-127 (PLF-127). (**B**) Zeta potential of IMC in IMC-NPs, IMC-NC/PLF5, IMC-NC/PLF10, and IMC-NC/PLF15. (**C**,**D**) Viscosity of IMC-NPs, IMC-NC/PLF5, IMC-NC/PLF10, and IMC-NC/PLF15 at (**C**) 10 °C and (**D**) 37 °C. (**E**) Image of IMC-NPs, IMC-NC/PLF5, IMC-NC/PLF10, and IMC-NC/PLF15 at 10 °C and 37 °C. Compositions of each ophthalmic IMC-NC formulation shown in Table 1; *n* = 7–8; * *p* < 0.05 vs. IMC-MC for each category, ^#^ *p* < 0.05 vs. PLF0 (IMC-NPs). The addition of PLF-127 did not affect IMC solubility and zeta potential in IMC-NC-incorporating ISG. The viscosity of ophthalmic PLF-127-based IMC-NC-incorporating ISG was higher at 37 °C than at 10 °C. In particular, the gelation of IMC-NC/PLF10 and IMC-NC/PLF15 was promoted at 37 °C, with viscosity of approximately 9.2 and 22.3 mPa∙s, respectively.

We previously showed that IMC-NCs could be attained via the treatment of a bead mill with MC [20,21], whereas the addition of 2-hydroxypropyl-β-cyclodextrin (HPCD) enhanced the stability of nanosuspensions [20]. Therefore, we attempted to prepare ophthalmic dispersions containing IMC-NCs (IMC-NPs), according to these previous studies. Figure 1 shows the particle-size frequencies and atomic force microscopy (AFM) images of IMC in the ophthalmic IMC-NC formulations (IMC-NPs and IMC-NC-incorporating ISG) shown in Table 1. IMC nanoparticulation was achieved through the bead-mill treatment, and drug-particle size in the IMC-NPs was 50–170 nm. The drug-particle size of the IMC-NC-incorporating ISG was also similar to that in IMC-NPs, although the peak of 130 nm IMC particles was decreased with PLF-127, with a shift to 100–170-nm particles. Figure 2A shows IMC solubility in ophthalmic IMC-NC formulations. IMC solubility was enhanced by nanoparticulation, and IMC solubility in the IMC-NC formulations with the bead mill was approximately 5.2-fold higher than that in dispersions containing IMC microcrystals (IMC-MCs) without the bead mill. On the other hand, PLF-127 did not affect solubility, since IMC solubility was similar between IMC-NPs and the IMC-NC-incorporating ISG for each category. Insolubilized IMC accounted for approximately 93% of the ophthalmic IMC-NC formulation. Zeta potentials describe the stability of suspensions. Therefore, we measured the zeta potentials of the IMC-NC-incorporating ISG (Figure 2B). The zeta potentials of the ophthalmic IMC-NC formulations with or without PLF-127 were approximately −41–45 mV. Furthermore, we investigated changes in the viscosity of the ophthalmic IMC-NC formulations. Viscosity was enhanced with PLF-127 at 10 °C, and the viscosity of IMC-NCs with 5 g of PLF-127 (IMC-NC/PLF5), IMC-NC/PLF10, and IMC-NC/PLF15 was approximately 1.3-, 1.7-, and 2.2-fold higher, respectively, than that of IMC-NPs (Figure 2C). On the other hand, gelation of the IMC-NC-incorporating ISG was observed at 37 °C, and viscosity was remarkably increased (Figure 2D). In particular, IMC-NC/PLF10 and IMC-NC/PLF15 were in a gel state at 37 °C, in contrast to the state of IMC-NC/PLF5 (Figure 2E).

### 2.2. Stability of Ophthalmic IMC-NC Formulations

**Figure 3:**
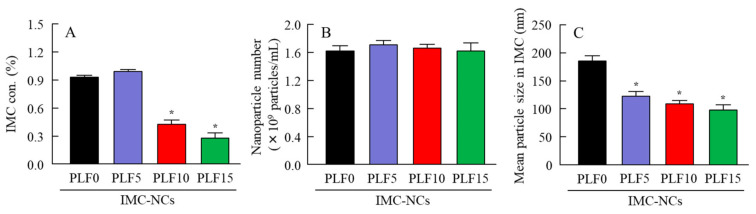
Dispersibility, nanoparticle number, and size of IMC in IMC-NPs, IMC-NC/PLF5, IMC-NC/PLF10, and IMC-NC/PLF15 one month after the preparation. Ophthalmic IMC-NC formulations stored at 20 °C for one month. (**A**) IMC concentration at 5 mm under surface of each ophthalmic IMC-NC formulation stored for one month (dispersibility). IMC (**B**) nanoparticle number and (**C**) size in each ophthalmic IMC-NC formulation one month after preparation. (**B**,**C**) Ophthalmic IMC-NC formulations were gently stirred before measurement. Compositions of each ophthalmic IMC-NC formulation shown in Table 1; *n* = 5; * *p* < 0.05 vs. PLF0 (IMC-NPs) for each category. For IMC-NPs and IMC-NC/PLF5, IMC-NCs were evenly dispersed throughout, although in IMC-NC/PLF10 and IMC-NC/PLF15, the upper layer was poor in IMC-NCs and the lower layer was rich in IMC-NCs at one month after preparation. On the other hand, PLF-127 enhanced the stability of IMC-NCs.

Suspensions without suitable additives easily aggregate. Therefore, we investigated whether the dispersibility, particle number, and size frequency of IMC in the IMC-NC-incorporating ISG changed one month after preparation (Figure 3). IMC in IMC-NPs and IMC-NC/PLF5 was evenly dispersed throughout, with an IMC concentration in the upper layer of approximately 1%. In contrast to the results for IMC-NC/PLF5, the concentration of IMC in the upper layer of IMC-NC/PLF10 and IMC-NC/PLF15 was poor at one month after preparation (Figure 3A). On the other hand, the aggregation and precipitation of IMC-NCs were not observed in the ophthalmic IMC-NC formulations, with or without PLF-127 (Figure 3B). Moreover, the stability of IMC-NCs in IMC-NPs was enhanced by the addition of PLF-127, since the mean particle size of IMC in the IMC-NC-incorporating ISG was higher than that of the IMC-NPs (Figure 3C).

### 2.3. Release of IMC from Ophthalmic IMC-NC Formulations

**Figure 4:**
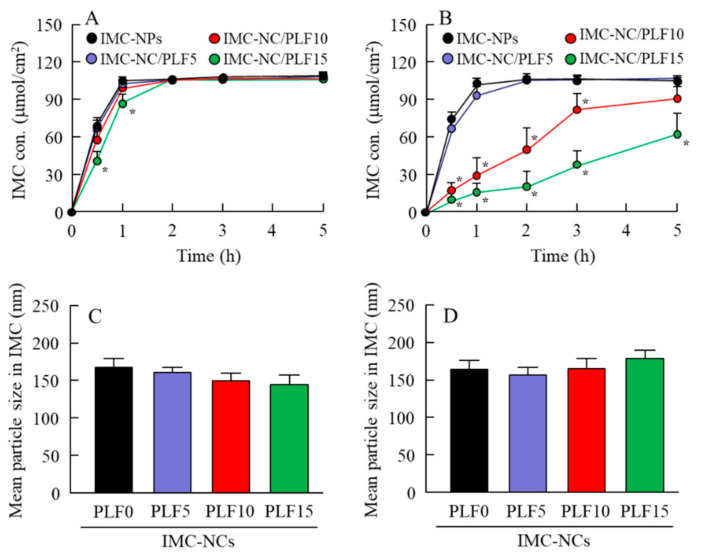
Diffusion of IMC-NCs in each ophthalmic IMC-NC formulation through methacrylate cell. Diffusion behavior of IMC-NCs in IMC-NPs, IMC-NC/PLF5, IMC-NC/PLF10, and IMC-NC/PLF15 at (**A**) 10 °C and (**B**) 37 °C. Mean particle size of IMC in reservoir chamber of methacrylate cell with each applied ophthalmic IMC-NC formulation at (**C**) 10 °C and (**D**) 37 °C. The compositions of each ophthalmic IMC-NC formulation are shown in Table 1; *n* = 8; * *p* < 0.05 vs. IMC-NPs for each category. The diffusion of IMC-NCs decreased with PLF-127. In particular, the release of IMC-NCs from gelled IMC-NC/PLF10 and IMC-NC/PLF15 at 37 °C was lower in comparison with IMC-NC without PLF-127 (IMC-NPs).

Next, we evaluated the effect of enhanced viscosity on drug diffusion and release in the IMC-NC-incorporating ISG (Figure 4). IMC in the IMC-NPs shifted to the reservoir side from the donor side at 10 °C and 37 °C, while IMC particle size remained in the nanosize range in the reservoir chamber. Although the behavior of IMC release in the IMC-NC-incorporating ISG with 5% and 10% PLF-127 was similar to that in IMC-NPs, the time needed for IMC transfer to the reservoir side from the donor side was increased in the IMC-NC-incorporating ISG with 15% PLF-127 (IMC-NC/PLF15, Figure 4A). Otherwise, no significant difference was observed in the release of IMC between IMC-NPs and IMC-NC/PLF5 at 37 °C. However, the diffusion of IMC-NCs decreased upon the addition of 10% and 15% PLF-127, while the release of IMC-NCs from gelled IMC-NC/PLF10 and IMC-NC/PLF15 was significantly attenuated compared to that from IMC-NPs (Figure 4B). On the other hand, IMC particles released from the IMC-NC-incorporating ISG remained in the nanosize range in the reservoir chamber at both 10 °C and 37 °C (Figure 4C,D).

### 2.4. Drug Behavior in Rabbits Instilled with IMC-NC-Incorporating ISG

**Figure 5:**
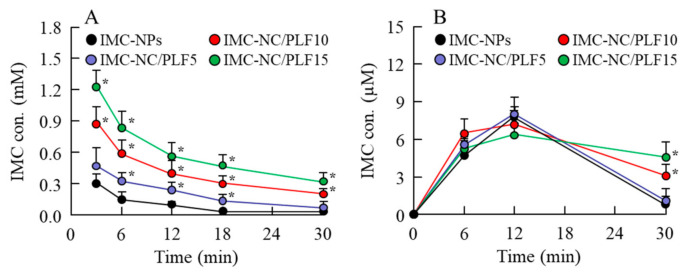
IMC levels in (**A**) lacrimal fluid (LF) and (**B**) blood of rabbits instilled with each ophthalmic IMC-NC formulation. Compositions of each ophthalmic IMC-NC formulation shown in Table 1; *n* = 6–8; * *p* < 0.05 vs. IMC-NPs for each category. Retention time of IMC levels in rabbit LF was enhanced with PLF-127 in IMC-NC-incorporating ISG. Addition of PLF-127 delayed IMC elimination in blood after instillation.

**Figure 6:**
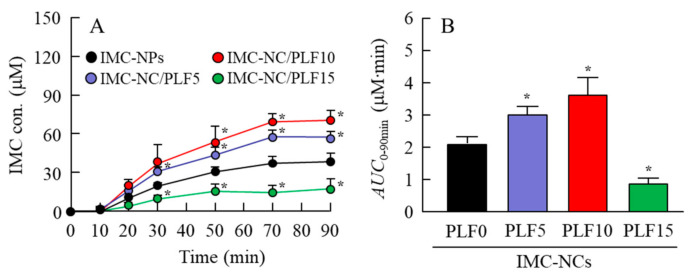
In vivo transcorneal penetration of each ophthalmic IMC-NC formulation. (**A**) Changes in IMC levels in rabbit LF instilled with each ophthalmic IMC-NC formulation. (**B**) Area under drug concentration–time curve between 0 and 90 min *(AUC*_0–90min_) in aqueous humor of rabbits instilled with each ophthalmic IMC-NC formulation. Compositions of each ophthalmic IMC-NC formulation shown in Table 1; *n* = 5–8; * *p* < 0.05 vs. IMC-NPs for each category. Introduction of 5% and 10% PLF-127 increased IMC transcorneal penetration in rabbits instilled with IMC-NC-incorporating ISG. In contrast to results for optimal PLF-127 (5% and 10%), excessive PLF-127 (15%) decreased IMC-NC uptake after instillation.

Figure 5 shows the IMC content in the lacrimal fluid (LF) and blood of rabbits instilled with the IMC-NC-incorporating ISG. The IMC content in the LF of rabbits instilled with IMC-NPs flowed from the ocular surface for 18 min. However, the retention time of IMC levels in the LF of rabbits was enhanced with PLF-127 in the IMC-NC-incorporating ISG, and the IMC content in the LF of rabbits instilled with IMC-NC/PLF5, IMC-NC/PLF10, and IMC-NC/PLF15 was approximately 2.3-, 4.1-, and 5.9-fold higher, respectively, than that with IMC-NPs 6 min after instillation (Figure 5A). Moreover, the addition of PLF-127 delayed the elimination of IMC in the blood after instillation (Figure 5B). Figure 6 shows the permeation of IMC in the IMC-NC-incorporating ISG, and Table 2 summarizes the pharmacokinetic parameters calculated from the in vivo transcorneal penetration data. The addition of 5% and 10% PLF-127 significantly enhanced the permeation of IMC in comparison with IMC-NPs. Excessive PLF-127 (15%) also prolonged IMC residence time in the LF after instillation, whereby the residence time was higher than that in IMC-NC/PLF5 and IMC-NC/PLF10. However, IMC content and *k*_a_ in the aqueous humor of rabbits instilled with IMC-NC/PLF15 was significantly lower than that with ophthalmic IMC-NC formulations with 0–10% PLF-127 (Figure 6). Furthermore, it was important to evaluate the corneal toxicity of the IMC-NC-incorporating ISG. Therefore, we examined whether the rabbit cornea was damaged by repetitive instillation of the IMC-NC-incorporating ISG for one month (twice a day). As a result, corneal toxicity was not observed in the rabbits repetitively instilled with the IMC-NC-incorporating ISG for one month.

## 3. Discussion

It is desirable that drugs topically administered to the eye are rapidly absorbed and lead to a therapeutic effect. However, traditional eye drops cannot provide and maintain an adequate drug concentration in the intraocular tissue, such as the iris, lens, and retina, and it is important to resolve this conundrum and its shortcomings. Therefore, many endeavors were made to enhance ocular BA via prolongation of the drug residence time on the cornea and an improvement of the drug corneal penetration, which is the major route of drug entry into the internal eye [30]. However, further improvements are necessary to extend the drug residence time in the ocular surface, and to increase delivery into the intraocular area after instillation for the effective therapy of ocular diseases. In this study, we designed an IMC-NC-incorporating ISG (IMC-NC/PLF) and found that the combination of IMC-NCs and 5–10% PLF-127 as an in situ gelling system extended drug residence time on the cornea and improved ocular BA.

Nonsteroidal anti-inflammatory drugs (NSAIDs) are employed in the treatment of uveitis, postoperative inflammation, diabetic retinopathy, and age-related macular degeneration [31]. IMC is an NSAID, and it is difficult to formulate topical IMC ophthalmic solution since IMC has poor solubility, stability, and ocular BA [32,33]. Therefore, topical ophthalmic solutions of IMC are not marketed in Japan and the United States. On the other hand, Claudia et al. showed that IMC ophthalmic suspension (Indom^TM^, Alfa-Intes) had ocular distribution reaching relevant retinal IMC levels, and suggesting that this formulation may be useful in clinical practice for macular edema treatment [34]. Moreover, in the ongoing clinical trials, it was showed that IMC is at least as effective as ketorolac for the prevention of ocular inflammation following cataract surgery (https://clinicaltrials.gov/ct2/show/NCT00904904). We also reported that ophthalmic dispersions containing IMC-NCs (IMD-NPs) showed high corneal penetration via multiple energy-dependent endocytoses [21]. Therefore, it is possible that the development of DDSs based on IMC-NCs may improve problems such as poor ocular BA. As a result of this, we selected IMC to evaluate the ophthalmic PLF-127-based IMC-NC-incorporating ISG in this study.

First, we designed the IMC-NC-incorporating ISG. We previously reported that 0.5% MC is essential for the preparation of drug NCs via the bead-mill method, since IMC becomes meringuelike when subjected to the bead-mill method without MC. According to these previous results, IMC nanoparticles were obtained via the bead mill method with MC [20,21]. Moreover, benzalkonium chloride (BAC) was used as a preservative, and mannitol was added to attenuate the corneal toxicity of BAC. In addition, IMC-NCs were coated with HPCD to prevent cohesion and aggregation. In this study, we selected these additives to prepare the IMC-NPs, and the drug-particle size of the IMC-NPs was 50–170 nm (Figure 1). It was known that the solubility was enhanced by the nanoparticulation. Therefore, we evaluated the solubility of IMC-NCs by comparing them with the solubility of IMC-MCs. Although the solubility of IMC in the IMC-NC formulations was approximately 5.2-fold higher than that of dispersions containing IMC microcrystals (IMC-MCs), insolubilized IMC accounted for approximately 93% of the ophthalmic IMC-NC formulations (Figure 2A). These results showed that ophthalmic dispersions containing IMC-NCs can be prepared using the protocol demonstrated in this study, whereby IMC solubility was enhanced by nanoparticulation.

Next, we attempted to prepare the ophthalmic PLF-127-based IMC-NC-incorporating ISG, and we investigated the effect of PLF-127 on the characteristics of the IMC-NC-incorporating ISG. Edsman et al. [29] reported that PLF-127 concentration strongly affects the phase-transition temperature, and, in their preparation of an ISG, PLF-127 was used in concentrations above approximately 20–23%. However, the phase-transition temperature of a PLF-127 solution is under 25 °C; thus, the PLF-127 solution would already become a gel at room temperature. Therefore, it is difficult to instill ophthalmic formulations containing 20–23% PLF-127. Moreover, it may cause some patient discomfort, as 20–23% PLF-127 eye drops may show corneal toxicity, whereas a concentration of 16% or lower of PLF-127 was reported to be safer [29]. In addition, a nonchemically crosslinked hydrogel was formed with 18% or greater PLF-127 solution upon warming to ambient temperature [29]. From these reports, we established the concentration of PLF-127 as 5–15% in this study. Although the addition of PLF-127 did not affect the solubility and zeta potential of IMC in the IMC-NC-incorporating ISG, the 130 nm IMC aggregation was decreased with PLF-127 (Figure 1 and Figure 2). On the other hand, gelation of IMC-NC/PLF10 and IMC-NC/PLF15 was observed at 37 °C, whereas IMC-NC/PLF5 remained in solution (Figure 2C–E). In general, viscosity enhancement is related to the aggregation and dispersibility of suspensions. Therefore, we measured changes in aggregation and dispersibility in the IMC-NC-incorporating ISG. The addition of PLF-127 attenuated the aggregation and precipitation of IMC-NCs (Figure 3B,C). IMC-NCs in the IMC-NPs and IMC-NC/PLF5 were evenly dispersed throughout, although IMC concentration in the upper layer of IMC-NC/PLF10 and IMC-NC/PLF15 was poor at one month after preparation (Figure 3A). These results showed that 5% or less PLF-127 is suitable to maintain high dispersibility in the IMC-NC-incorporating ISG. In addition, no significant difference was observed in the release of IMC between IMC-NPs and IMC-NC/PLF5 at 37 °C; however, the release of IMC-NCs from gelled IMC-NC/PLF10 and IMC-NC/PLF15 was significantly attenuated, compared to that from IMC-NPs at 37 °C (Figure 4). The viscosity of IMC-NC/PLF10 and IMC-NC/PLF15 was high, and these formulations gelled slightly at 10 °C (Figure 2C–E). This gelled state may prevent even dispersion, drug diffusion, and the release of nanoparticles, with these results suggesting that 5% PLF-127 is suitable to formulate the IMC-NC-incorporating ISG from the viewpoint of dispersion stability.

**Figure 7:**
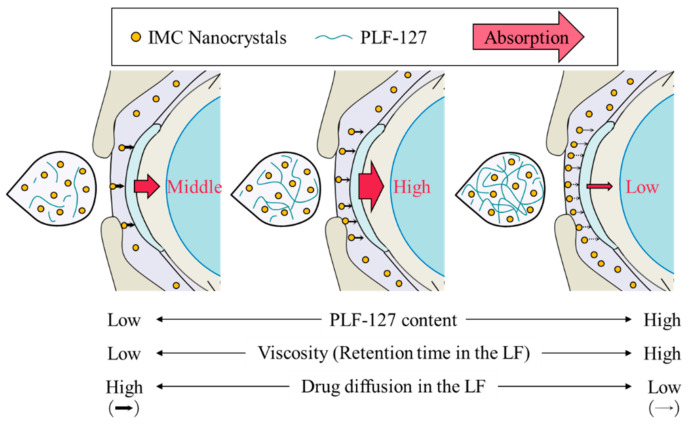
Effect of PLF-127 content on corneal permeability in IMC-NC-incorporating ISG.

Thermosensitive ISGs, such as PLF-127, have solution–gel transition properties, and they can prolong drug residence time on the cornea by converting the eye-drop formulations from a solution type to a gel upon instillation [35,36]. We also measured corneal toxicity and IMC content in the LF and aqueous humor of rabbits instilled with the IMC-NC-incorporating ISG. Corneal toxicity was not observed in rabbits repetitively instilled with the IMC-NC-incorporating ISG for one month, and the retention time of IMC levels in the LF of rabbits was enhanced with PLF-127 in the IMC-NC-incorporating ISG (Figure 5A). On the other hand, formulations with 5% and 10% PLF-127 increased the transcorneal penetration of IMC in rabbits instilled with the IMC-NC-incorporating ISG, although excessive PLF-127 (15%) decreased the uptake of IMC-NCs after instillation (Figure 5A). We previously reported the 2 pathway was related the corneal penetration of IMC-NCs. One pathway is that IMC-NCs were attached onto the cornea after the instillation of ophthalmic dispersions containing IMC-NCs (IMC-NPs), and dissolved IMC in the formulation penetrate through the cornea and be released into the aqueous humor [20,21]. Another pathway is that the IMC-NCs are taken up into the corneal epithelium by energy-dependent endocytosis (CavME, CME and MP pathways), especially CavME, and cross to the corneal stromal side. From there, the IMC-NCs are dissolved in the cornea and released into the aqueous humor in a soluble form. From the 2 pathways, an enhancement of BA in the IMC-NCs was led [21]. In addition, Kim et al. highlight the importance of formulations that conform to the ocular surface before viscosity enhancement for increased and prolonged ocular surface contact and drug absorption [37]. Thus, the ratio of attachment in corneal tissue to the corneal penetration of IMC in IMC-NC/PLF15, and the ratio of attachment of IMC-NCs and corneal tissue may be low in comparison with other IMC-NC-incorporating ISG with 5% and 10% PLF-127, since the release and the diffusion of IMC-NCs from IMC-NC/PLF15 were significantly reduced compared to ophthalmic IMC-NP formulations with and without 0–10% PLF-127 (Figure 4). Taken together, we hypothesize that enhanced viscosity prolongs the precorneal resident time of IMC-NCs. On the other hand, the diffusion of IMC-NCs into the LF was decreased while viscosity was increased, resulting in corneal permeation and enhanced drug BA (Figure 7). Further studies using medium-sized animals, such as rabbits, pigs, and monkeys, are needed to elucidate the usefulness of the PLF-127-based IMC-NC-incorporating ISG, since organizational structure and drug behavior in the eye are different in rats and humans.

## 4. Materials and Methods

### 4.1. Animals

Rabbits (2.5–3.0 kg) were purchased from Shimizu Laboratory Supplies Co., Ltd. (Kyoto, Japan) and housed under standard conditions. A commercial diet (CR-3) was provided by Clea Japan Inc. (Tokyo, Japan), and rabbits were allowed free access to the CR-3 and water. The rabbits used in the experiments were approved by Kindai University on 1 April 2013 (project identification code KAPS-25-004). All experiments were performed in accordance with the ARVO animal guidelines.

### 4.2. Chemicals

IMC, isoflurane, mannitol (d-mannitol), and acetic acid were purchased from Wako Pure Chemical Industries, Ltd. (Osaka, Japan). PLF-127 was provided by Funakoshi Co., Ltd. (Tokyo, Japan), and HPCD was kindly donated by Nihon Shokuhin Kako Co., Ltd. (Tokyo, Japan). Schirmer tear test strips were purchased from AYUMI Pharmaceutical Corporation (Tokyo, Japan). Oxybuprocaine hydrochloride (Benoxil^®^ ophthalmic solution 0.4%) was obtained from Santen Pharmaceutical Co., Ltd. (Osaka, Japan). SM-4-type MC was supplied by Shin-Etsu Chemical Co., Ltd. (Tokyo, Japan), and pentobarbital was provided by Sumitomo Dainippon Pharma Co., Ltd. (Toyo, Japan). BAC, methanol, and acetonitrile were purchased from Kanto Chemical Co., Inc. (Tokyo, Japan). Fluorescein was obtained from Alcon (Tokyo, Japan). All other used chemicals were of the highest commercially available purity.

### 4.3. Preparation of Ophthalmic IMC-NC Formulations

IMC-NPs were prepared following our previous reports [18,19,20,21]. Briefly, commercially available IMC powder (microcrystalline) and MC powder were mixed in a 1.5 mL tube with 1 mm zirconia beads at 3000 rpm for 30 s at 4 °C, using the Bead Smash 12 (Wakenyaku Co. Ltd., Kyoto, Japan). The mixture was dispersed in purified water containing BAC, mannitol, and HPCD, and then crushed with the Bead Smash 12 at 5500 rpm for 30 s at 4 °C. The mill treatment was repeated 30 times, after which the dispersions were crushed at 1500 rpm for 3 h with a Shake Master NEO (Bio-Medical Science Co., Ltd., Tokyo, Japan). The milled IMC dispersions were filtrated using a 0.22 µm pore membrane filter, and the filtrated dispersions containing IMC-NCs were used as IMC-NPs in this study. The IMC-NC/PLF formulations were prepared by adding a PLF-127 solution containing MC, BAC, mannitol, and HPCD to the IMC-NPs. The pH of ophthalmic IMC-NC formulations was adjusted to 5.5. Table 1 shows the composition of ophthalmic formulations containing IMC-NCs used in this study.

### 4.4. Characteristics of Ophthalmic IMC-NC Formulations

The characteristics of the ophthalmic IMC-NC formulations were measured following our previous reports [20,21,22,23]. Briefly, each ophthalmic IMC-NC formulation was stored in the dark at 20 °C for 30 days to measure IMC dispersibility, and samples were taken from 5 mm under the surface over time. Then, IMC content, particle size, and nanoparticle number in the collected samples were expressed as an index for dispersibility. IMC content was measured on an HPLC LC-20AT system (Shimadzu Corp. Kyoto, Japan) with an Inertsil^®^ ODS-3 column (GL Science Co., Inc., Tokyo, Japan) with detection at 254 nm. In the HPLC method, the mobile phase was acetonitrile/50 mM acetic acid (40%/60%, *v*/*v*) at a flow rate of 0.25 mL/min, and propyl *p*-hydroxybenzoate was used as an internal standard. Particle-size distributions of the IMC-NCs were analyzed using an SALD-7100 (Shimadzu Corp., Kyoto, Japan), and the refractive index to analyze IMC particles was set at 1.60–0.10i in this study. Moreover, a NANOSIGHT LM10 (QuantumDesign Japan, Tokyo, Japan) was used to evaluate the particle-size distribution and particle number of the IMC-NCs. In the measurement, viscosity was set to 1.27 mPa⋅s. IMC solubility was measured using a Beckman Optima^TM^ MAX-XP Ultracentrifuge (Beckman Coulter, Osaka, Japan) and the HPLC method. For the measurement of solubility, insolubilized IMC-NCs were removed from the ophthalmic IMC-NC formulations via centrifugation at 100,000× *g*, and the IMC content in the supernatants was measured using HPLC, as described above. Viscosity at 10 °C and 37 °C was analyzed using an Anton Paar MCR302 attached to a CP50-1 (Anton Paar Japan K.K, Tokyo, Japan). The measurement was performed 10 times, and the mean was used in this study. Measurement conditions were as follows: shear rate, 90–100 rpm/s; measurement time, 2 s; interval, 1 s. The AFM image of the IMC-NCs was created from a combination of phase and height images using an SPM-9700 (Shimadzu Corp., Kyoto, Japan). A Model 502 zeta-potential analyzer (Nihon Rufuto Co., Ltd., Tokyo, Japan) was used to analyze the zeta potential of ophthalmic IMC-NC formulations.

### 4.5. Diffusion of Ophthalmic IMC-NC Formulations

A 0.22 µm pore membrane filter was equipped to a methacrylate cell, and one side of the methacrylate cell (donor chamber) was filled with ophthalmic IMC-NC formulations, while the other side (reservoir chamber) was filled with phosphate-buffered saline. Experiments were performed at 10 °C and 37 °C. Fifty microliters of the sample was taken from the reservoir chamber over time and replaced with the same volume of saline. IMC content in the samples was measured using HPLC, as described above.

### 4.6. Corneal Toxicity of Ophthalmic IMC-NC Formulations

Thirty microliters of each ophthalmic IMC-NC formulation was repetitively instilled in rabbits twice per day (9:00 and 19:00) for one month. Then, 30 µL of 1% fluorescein was instilled, and the wound area in the cornea and the presence of hyperemia were monitored using a TRC-50X (Topcon, Tokyo, Japan). The 1% fluorescein was prepared by saline.

### 4.7. Changes in IMC Content in LF and Blood

Thirty microliters of each ophthalmic IMC-NC formulation was instilled, and LF and blood were collected. A Schirmer tear test strip was used to collect the LF, and the IMC was extracted from the LF in the Schirmer tear test strips. The blood was collected from the vena cava and centrifuged to provide serum before using the samples to measure the IMC. IMC content was measured using HPLC, as described above.

### 4.8. Transcorneal Penetration of Ophthalmic IMC-NC Formulations

Rabbits were anesthetized using isoflurane and a topical anesthetic (Benoxil^®^ ophthalmic solution 0.4%), and the various formulations were instilled into each eye 3 min before sampling the aqueous humor. Five microliters of aqueous humor was collected over time, and the IMC concentration in the aqueous humor was determined using HPLC, as described above. The area under the drug concentration–time curve between 0 and 90 min (*AUC*_0–90min_) in the aqueous humor was determined according to the trapezoidal rule, up to the last indomethacin concentration measurement point (90 min). The IMC concentration data in the aqueous humor after a single injection of 20 µL of IMC solution into the anterior chamber of the eye were analyzed according to Equation (1):

(1) $$C_{\mathrm{AH}}=C_{0}\cdot e^{-k_{e}\cdot t}$$

where *C*_AH_ is the IMC concentration in the aqueous humor at time *t*, *C*_0_ is the initial concentration of IMC in the aqueous humor, and *k*_e_ is the elimination rate constant of IMC from the aqueous humor. The *k*_e_ obtained in 5 experiments was 0.0511 min^−1^. The IMC concentration data in the aqueous humor after the instillation of 30 µL of each ophthalmic IMC-NC formulation were analyzed according to Equation (2):

(2) $$C_{\mathrm{AH}}=\frac{k_{a}\cdot F\cdot X}{V_{d}\cdot \left(k_{a}-k_{e}\right)}(e^{-k_{e}\cdot \left(t-\tau \right)}-e^{-k_{a}\cdot \left(t-\tau \right)})$$

where *X* is the dose of the ophthalmic IMC-NC formulation, *k*_a_ is the absorption rate constant, *V*_d_ is the distribution volume (volume of anterior chamber, ca. 150 µL), *F* is the fraction of IMC absorption, and *τ* is the lag time.

### 4.9. Statistical Analysis

One-way analysis of variance (ANOVA) followed by Dunnett’s multiple comparison was used for statistical analysis, and a minimal *p*-value of 0.05 was chosen as the significance level (*p* < 0.05). Sample numbers (*n*) are shown in the figure legends. Data are expressed as mean ± standard error (SE).

## 5. Conclusions

We formulated an IMC-NC-incorporating ISG using PLF-127 (IMC-NC/PLF). We found that IMC-NCs with an optimal amount of PLF-127 (5–10%) resulted in higher IMC corneal permeation after instillation than the IMC-NPs with excessive PLF-127 did, probably because of the balance between higher residence time and faster diffusion of IMC-NCs on the ocular surface. In addition, from the viewpoint of drug stability and ocular BA, we showed that 5% PLF-127 was optimal for the preparation of the IMC-NC-incorporating ISG. These findings provide significant information for developing ophthalmic nanomedicines.

## Figures and Tables

**Table 1:** Composition of ophthalmic IMC-NC formulations.

Formulation	IMC	PLF	MC	BAC	Mannitol	HPCD	Purified Water	Treatment
IMC-NPs	1 g	—	0.5 g	0.001 g	0.1 g	5 g	100 g	Bead mill
IMC-NC/PLF5	1 g	5 g	0.5 g	0.001 g	0.1 g	5 g	100 g	Bead mill
IMC-NC/PLF10	1 g	10 g	0.5 g	0.001 g	0.1 g	5 g	100 g	Bead mill
IMC-NC/PLF15	1 g	15 g	0.5 g	0.001 g	0.1 g	5 g	100 g	Bead mill

HPCD, 2-hydroxypropyl-β-cyclodextrin; MC, methylcellulose; BAC, benzalkonium chloride.

**Table 2:** Pharmacokinetic parameters for the in vivo transcorneal penetration of each ophthalmic IMC-NC formulation.

Formulation	IMC-NPs	IMC-NC/PLF5	IMC-NC/PLF10	IMC-NC/PLF15
*k*_a_ (×10^−4^/min)	23.7 ± 5.5	41.4 ± 7.9 *	46.8 ± 8.1 *	18.0 ± 4.8
*r* (min)	7.34 ± 0.84	7.48 ± 0.93	7.44 ± 0.85	7.56 ± 1.09

Parameters were calculated according to Equations (1) and (2) (see Materials and Methods). Compositions of each ophthalmic IMC-NC formulation shown in Table 1; *n* = 5–8; * *p* < 0.05 vs. IMC-NPs for each category.

## Abbreviations

ANOVA	one-way analysis of variance
AFM	atomic force microscopy
*AUC*_0–90min_	area under drug concentration–time curve between 0 and 90 min
BA	bioavailability
BAC	benzalkonium chloride
CavME	caveolae-dependent endocytosis
CME	clathrin-dependent endocytosis
DDS	Drug-delivery systems
HPCD	2-hydroxypropyl-β-cyclodextrin
IMC	indomethacin
IMC-MCs	dispersions containing indomethacin microcrystals
IMC-NCs	indomethacin nanocrystals
IMC-NC/PLF	in situ gel incorporating indomethacin nanocrystals with PLF-127
IMC-NPs	ophthalmic dispersions containing indomethacin nanocrystals
ISG	in situ gel
LF	lacrimal fluid
MC	methylcellulose
MP	micropinocytosis
NCs	nanocrystals
NPs	nanoparticles
NSAIDs	nonsteroidal anti-inflammatory drugs
PLF-127	Pluronic F-127
SE	standard error
